# Modulation of Tumor Immunity by Soluble and Membrane-Bound Molecules at the Immunological Synapse

**DOI:** 10.1155/2013/450291

**Published:** 2013-03-07

**Authors:** Pablo A. González, Leandro J. Carreño, Pablo F. Céspedes, Susan M. Bueno, Claudia A. Riedel, Alexis M. Kalergis

**Affiliations:** ^1^Millennium Institute on Immunology and Immunotherapy, Departamento de Genética Molecular y Microbiología, Facultad de Ciencias Biológicas, Pontificia Universidad Católica de Chile, Avenida Libertador Bernardo O'Higgins no. 340, Santiago 8331010, Chile; ^2^INSERM U1064, Nantes, France; ^3^Millennium Institute on Immunology and Immunotherapy, Departamento de Ciencias Biológicas, Facultad de Ciencias Biológicas y Facultad de Medicina, Universidad Andrés Bello, Chile; ^4^Departamento de Reumatología, Facultad de Medicina, Pontificia Universidad Católica de Chile, Chile

## Abstract

To circumvent pathology caused by infectious microbes and tumor growth, the host immune system must constantly clear harmful microorganisms and potentially malignant transformed cells. This task is accomplished in part by T-cells, which can directly kill infected or tumorigenic cells. A crucial event determining the recognition and elimination of detrimental cells is antigen recognition by the T cell receptor (TCR) expressed on the surface of T cells. Upon binding of the TCR to cognate peptide-MHC complexes presented on the surface of antigen presenting cells (APCs), a specialized supramolecular structure known as the immunological synapse (IS) assembles at the T cell-APC interface. Such a structure involves massive redistribution of membrane proteins, including TCR/pMHC complexes, modulatory receptor pairs, and adhesion molecules. Furthermore, assembly of the immunological synapse leads to intracellular events that modulate and define the magnitude and characteristics of the T cell response. Here, we discuss recent literature on the regulation and assembly of IS and the mechanisms evolved by tumors to modulate its function to escape T cell cytotoxicity, as well as novel strategies targeting the IS for therapy.

## 1. Introduction

Human beings are constantly exposed to xenobiotics and microbes that can alter normal cell physiology and thus, potentially lead to tumor growth and cancer [1–5]. Currently, cancer is a leading cause of death worldwide accounting nearly for 13% of all deaths in 2008 (World Health Organization, WHO, http://www.who.int/en/). Noteworthy, recent projections predict that cancer-related deaths will continue rising to estimated 13.1 million deaths in 2030 (WHO, http://www.who.int/en/). To avoid transformed cells from expanding into the organism and causing pathology, effective surveillance by the adaptive immune response continuously needs to take place [6–9]. An essential element determining the balance between immunity and tolerance is antigen recognition on the surface of antigen presenting cells (APC) by the T cell receptor (TCR) on T cells [10–19]. T cells constantly scan the APC surface searching for antigens to activate and exert their effector functions. Identification of T cell antigens on the surface of APCs will lead to the rearrangement of intracellular and extracellular molecules at the T cell APC interface, ultimately leading to the assembly of a specialized supramolecular structure known as the immunological synapse (IS) [20, 21]. Importantly, the characteristics of the assembled IS will determine the fate of T cells and their capacity to clear malignant cells [2, 7, 22–26]. Here we discuss recent literature on the role of IS assembly and its modulation in tumor immunity.

## 2. Tumor Antigens

 Malignant cells that lead to tumor growth and cancer can derive from tissue injury, cell stress, aging, and pathogenic microbes that transform the genetic and physiological properties of normal cells [27]. During this process, transformed cells become modified in such a way that they acquire increased replication fitness and resistance to the immune system [28, 29]. Importantly, because malignant cells are predisposed to accumulate genetic mutations, these cells will create novel genetic polymorphisms [30]. These genetic mutations may translate into new amino acid sequences at the protein level that could be recognized by T and B cells as antigenic ligands [30, 31]. Noteworthy, transformed cells will also express sets of genes that were originally turned off in the parental cell [32–35]. Thus, malignant cells derived from nonimmunological cells may acquire the capacity to express and secrete immune-derived molecules, such as membrane-bound immune-modulatory molecules and cytokines that will modify immune cells for their benefit [32–36]. Furthermore, transformed cells may evolve to halt the expression of genes that favor immune surveillance, thus escaping immune checkpoint [32, 37]. For instance, most tumor cells reduce the expression of major histocompatibility complex molecules needed for natural killer and T-cells recognition [32, 38, 39]. However, it has been observed that tumor cells are prone to express certain endogenous proteins at significantly increased levels as compared to normal cells, which increases the likelihood of antigens derived from these proteins to be exposed to the immune system [30, 40, 41]. These specific antigens are the basis for T cell-specific immunity to tumors (discussed below).

## 3. Tumor Recognition by T Cells 

**Figure 1:**
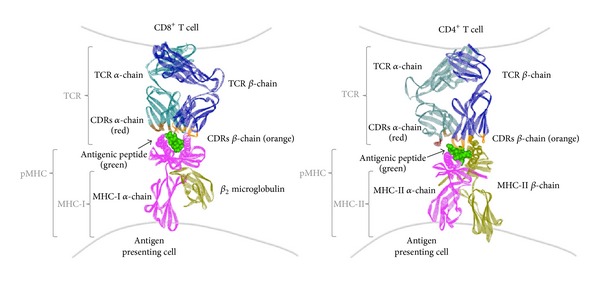
The structure of the T cell receptor (TCR) and its ligand, the peptide-MHC complex (pMHC). The TCR is a disulfide-bound heterodimer composed by one alpha (dark green) and one beta (blue) chain. Diversity within TCR molecules is mainly concentrated at the complementarity determining regions (CDRs, in red and orange) of the alpha and beta chains at the antigen-recognition region. Antigenic peptides are presented in the peptide groove of MHC molecules. MHC-I molecules are composed by an alpha chain (pink) that harbors the peptide-presentation groove and a small beta-2 microglobulin chain (yellow, *β*_2_ m). On the other hand, MHC-II molecules are composed by one alpha (pink) and one beta (yellow) chain. As shown for this molecule, the peptide-presentation groove in these molecules is formed by both chains. Antigenic peptides (green) have amino acids that are exposed to the TCR molecule and amino acids that are buried within the MHC groove. Left: the TCR/pMHC complex of a human melanoma-specific TCR (DMF5) bound to MHC-I HLA-A2 with the the MART-1 (26–35) peptide (RCSB Protein Data Bank accession number DOI: 10.2210/pdb3qdg/pdb, 3QDG). Right: the TCR/pMHC complex of a human melanoma-specific TCR (E8) bound to the MHC-II molecule HLA-DR1 and an epitope from mutant triosephosphate isomerase (RCSB Protein Data Bank accession number DOI: 10.2210/pdb4e41/pdb, 4E41). Both molecules were modeled using the software ViewerLite 5.0 from Accelrys Inc.

Although immune surveillance continuously restricts tumor growth in healthy individuals, transformed cells can ultimately overcome innate and adaptive immunity [37, 42]. T and B cells from adaptive immunity have been shown to play key roles in tumor immunity; these cells can be engaged to prevent and control tumorogenesis [43–47]. Although, antibodies against tumor antigens and immune-modulatory molecules have been shown to be helpful in tumor treatment [43, 48, 49], T cells are often involved in this process and have been shown to play significant roles in the control of proliferative malignant cells [48–51]. T cells recognize cognate antigens as small peptides bound to self-MHC molecules (pMHC complexes, Figure 1) [13, 15–19, 52, 53]. CD8^+^ T cells (CTLs) recognize antigenic peptides presented on MHC-I molecules, which are expressed on the surface of all nucleated cells in the host organism. On the other hand, CD4^+^ T cells (T helper, Th cells) recognize antigenic peptides in the context of MHC-II molecules, which are expressed on a smaller subset of cells, mainly at the surface of professional antigen presenting cells (APCs), such as dendritic cells (DCs) [54]. Similar to antibodies, TCR molecules display a tremendous diversity at the amino acid sequence level, mainly at the antigen-recognition region where amino acid sequence diversity is concentrated at specific sites within both the *α* and *β* chains (complementarity determining regions, CDRs) (Figure 1) [55–58]. It is estimated that TCR diversity nears 10^15^ combinations [59], allowing T cells to recognize unique pMHC complexes on the surface of APCs that derive from the processing and presentation of proteins from normal and transformed host cells, as well as from microbe proteins. 

**Figure 2:**
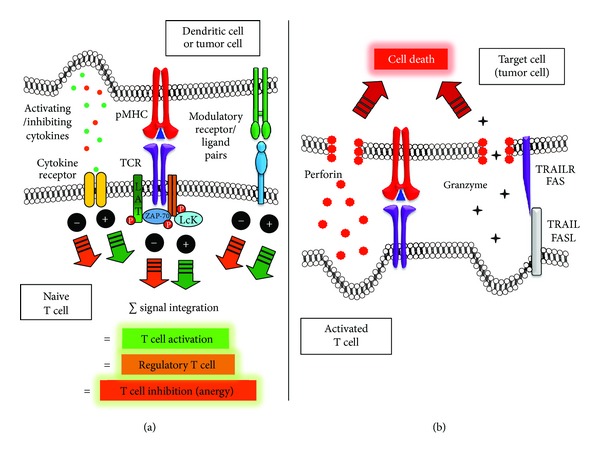
Activating and inhibiting immunological synapses for naïve and effector T cells. (a) APCs bear modulatory molecules on their surfaces that shape T cell activation and differentiation during IS assembly. Costimulatory molecules provide positive stimuli to T cells that are activating, while inhibitory molecules dampen T cell activation producing anergic or regulatory T cells. Tumor cells have been shown to express T cell inhibitory molecules on their surface to abrogate T cell activation or modulate their activity to produce inhibitory regulatory T cells. Cytokines and chemokines expressed by APCs or tumor cells can also modulate T cell activation synergizing or antagonizing with the above-mentioned membrane-bound molecules. Concentration of TCR/pMHC and adhesion molecules at the APC-T cell interface forms an immunological synapse. (b) CTLs exert their cytotoxic effects mainly through the release of soluble cell-killing molecules released towards target cells. Cytotoxic molecules include granule proteases known as granzymes aided by membrane-disruptive proteins known as perforins. Additionally, engagement of death receptors on the target cells, such as Fas (CD95) and TRAILR by FasL (CD95-ligand) and TRAIL molecules expressed on the surface of T cells, can also induce cell death mediated by apoptosis.

Recognition of cognate pMHC ligands on the surface of APCs can lead to a diverse array of T cell activation outcomes (Figure 2(a)). A common outcome is T cell activation and acquisition of killing capacity against target cells presenting surface antigens (Figure 2(b)). Although cytotoxic cell activity was initially thought to be a particular feature of cytotoxic T lymphocytes (CTLs), there is now supporting data that CD4^+^ Th cells can also display this activity, both against tumors and microbes [60–64]. Nevertheless, Th cells are mostly known for their effector functions exerted through the secretion of immune-modulatory cytokines that activate and shape the functions of other immune cells [9, 65, 66]. Notably, cytokines secreted by Th cells rely on their previous differentiation imprinted by professional APCs, such as dendritic cells (DCs) [67, 68]. Th cells are programmed to display particular phenotypes, mostly defined by the expression of transcription factors that commit the cells to secrete specific cytokine patterns or display regulatory functions. This has led to the identification of at least four Th subsets known as Th1, Th2, Th17, and regulatory T cells, which display individual features and mainly produce the signature cytokines IFN-*γ*, IL-4, IL-17, and IL-10, respectively [69].

For T cells to either exert their cytotoxic activities over tumor cells or secrete modulatory cytokines that affect directly or indirectly these malignant cells, antigen needs to be recognized on the surface of APCs. Upon T cell contact with APCs, TCRs scan the APC surface for antigen recognition. After antigenic pMHC complexes are recognized by TCRs, an intimate cell-cell interaction is established, which additionally involves the interaction of adhesion and modulatory molecules on the surface of both cells (Figure 2(a)). This tight cell-cell interaction leads to the assembly of an organized supramolecular structure known as the immunological synapse (IS). Noteworthy, the IS assembled between T cells and target cells (e.g., effector T cells interacting with tumor cells, Figure 2(b)) can significantly differ from the IS formed between T cells and professional antigen presenting cells (e.g., naive T cells interacting with DCs), because of membrane-bound and secreted molecules (Figure 2(a)). Here, we discuss these ISs in the context of tumor immunity.

## 4. Cytotoxic Immunological Synapse in Tumor Immunity

As discussed above, CTLs and Th cells can exert cytotoxic activities over target cells, such as tumor cells [60, 61, 64]. This cytotoxic activity can be mediated by the release of soluble cell-killing components by T cells towards malignant cells, such as granule proteases known as granzymes, which are aided by membrane-disruptive proteins named perforins (Figure 2(b)) [50, 70, 71]. Because these molecules are short lived, they require intimate cell interactions and hence IS assembly is needed between T cells and tumor cells for cytotoxicity to occur [72–74]. Additionally, engagement of death receptors on the tumor cells, such as Fas (CD95) and TRAILR by FasL (CD95-ligand) and TRAIL molecules expressed on the surface of T cells, can also induce cell death mediated by apoptosis [75]. Although Fas has been observed to localize at the IS between immortalized cells and T cells, the same has yet not been described for the TRAIL/TRAILR receptor pair [76]. However, localization of this receptor pair at the IS of T cells and tumor cells is likely to occur, as it would favor specific killing of tumor cells. 

Upon contact of T cells with APCs, TCRs scan the APC surface in search of cognate ligands forming initially a central ring of adhesion molecules with integrin pairs, such as LFA-1/ICAM-1, among others [20, 77, 78]. If cognate antigen is encountered, these adhesion molecules rapidly become surrounded by a ring of TCR/pMHC molecules, altogether defining an immature IS. Shortly after, this molecular pattern is inverted forming a central cluster of TCR/pMHC molecules known as central supramolecular activation cluster (cSMAC), which is surrounded by a peripheral ring of LFA-1/ICAM-1 molecules and is named the peripheral supra-molecular activation cluster (pSMAC) [20, 78–80], defining a mature IS. Importantly, this process depends on significant cytoskeleton rearrangements involving the microtubule organizing center (MTOC) and cytotoxic granules polarizing to the IS [81–83]. In fact, disruption of actin polymerization by cytochalasin D has been shown to disrupt IS formation [84, 85]. With the formation of IS, a tight and closer interaction is established between the T cell and the APC. Thus, large molecules such as the large glycoproteins CD43 and CD45 become excluded into a distal region termed dSMAC [86, 87]. Furthermore, CD45 has a tyrosine phosphatase domain that deactivates several targets within the T cell activating pathways upon ligand engagement, and thus its exclusion from the IS would favor positive signaling events within the T cell [88]. Although the large glycoprotein CD44 was thought to possess a similar localization at the dSMAC, more recent findings position this molecule directly at the IS, playing relevant roles both in cell-cell adhesion and the modulation of T cell activation [89]. Nevertheless, smaller molecules, such as the CD2–CD58 and CD2–CD48 pairs, are well known to remain within the synapse [87, 90, 91] and it is thought that interactions of these type of shorter molecules at the cSMAC could facilitate tight adhesions between T cells and APCs [88, 92]. This notion is supported by the observation that increasing the length of the CD2–CD48 complex can inhibit TCR engagement [93]. At this stage, early studies on the role of the IS suggested that these stable contacts formed at the IS would help initiating and maintaining signaling through TCRs by receptor aggregation [94–97]. However, it was later observed that initiation of TCR signaling, as determined by Ca^2+^ mobilization could occur at microclusters outside the cSMAC, even before IS had been formed [85, 98, 99]. This has now led to the notion that IS most likely plays a more prominent role in maintaining and then terminating TCR signaling, as well as polarizing cytokine secretion [100–102], rather than participating in early signaling events [98, 99, 103]. Although the role for IS is somewhat elusive, the importance of its assembly for T cell activation is unquestioned, as abrogation of this supramolecular organization can severely impair T cell activation [23, 104, 105]. In line with an important role for IS, several pathogens have evolved molecular mechanisms to impair or negatively modulate IS assembly and function [2, 24, 106, 107].

Upon TCR engagement by cognate pMHC complexes, phosphorilation of all three ITAM motifs in the CD3*ξ* molecule occurs by the kinases Lck and Fyn [108]. Phosphorylated CD3*ξ* molecules in turn recruit kinase ZAP-70, which associates to the phosphorylated ITAMs through its SH2 domains [108]. Once activated, ZAP-70 catalyzes phosphorylation of other molecules, such as the linker of activated T cells (LAT), which then leads to downstream signaling cascades resulting in an increase of cytoplasmic Ca^2+^ and the activation of transcription factors such as NF-AT, NF-*κ*B and AP-1 [109–114]. As mentioned above, these early signaling events can occur previous to IS assembly, taking place in microclusters before cSMAC formation. Although some microclusters will remain in the periphery, later in time most will coalescence to form the cSMAC. At this time, signaling molecules, such as ZAP-70 and SLP-76 will dissociate from the microclusters without migrating to the cSMAC [115–117].

## 5. Costimulation and Modulatory Molecules at the Immunological Synapse

Besides the engagement of TCRs by cognate pMHC ligands on the surface of APCs, T cells and APCs bear costimulatory molecules on their surfaces that modulate T cell activation and differentiation during IS assembly [9]. A group of costimulatory molecules that provide positive stimuli to T cells are those expressed by professional APCs, such as DCs, and include, within others, CD80 (B7.1), CD86 (B7.2), and the TNFR family members CD40, OX40L (CD134L/CD252), 4-1BBL (CD137L), and GITR-L, which interact with the ligands CD28 (binds both CD80 and CD86), CD40L, OX40 (CD134), 4-1BB (CD137), and GITR, respectively, which are expressed on the T cell surface [118–129]. Most of these costimulatory molecules are known to accumulate at the IS during antigen recognition and can provide additional positive stimuli to naïve T cells for activation, altogether defining a differentiated phenotype to T cells (Figure 2(a)) [9]. For instance, costimulatory molecules will enhance T cell clonal expansion and differentiation either because of increased rates of T cell proliferation or increased survival of the activated T cells [130–134]. 

However, professional APCs also express on their surface modulatory molecules that deliver inhibitory stimuli within T cells. Such molecules can regulate the activity of T cells in such a way to downregulate their activity and generate regulatory and anergic T cells. Molecules with these properties include B7-H1 (PD-L1; CD274), B7-DC (PD-L2; CD273), and ICOSL expressed on the APC surface [135–140]. These molecules bind, among others, PD-1 (binds to PD-L1 and PD-L2) and ICOS, respectively, on the T cell surface (Figure 2(a)). 

Because malignant cells would benefit from reduced cytotoxic T cell activity, several tumors and tumor cell lines express such molecules. This is the case for tumor cells within glioblastoma [141], hepatocellular carcinoma [142], colorectal carcinoma [143], breast cancer [144], non-Hodgkin lymphomas [145], lung cancer [146], and melanoma cells [34, 147], among others. These tumors have been shown to express T cell inhibitory molecules such as B7-H1 and ICOSL to engage their inhibitory counterparts on the T cell surface (Figure 2(a)). On the contrary, tumors that are induced to express costimulatory molecules that activate T cells, such as B7.1 (also known as CD80), and B7.2 (also known as CD86) are cleared by the immune system and thus may be used as a strategy to promote tumor immunity [148, 149].

Other inhibitory molecules, besides the ones described above, may also participate in T cell-APC IS dysfunction, such as CD200, CD270, and CD276 [150]. These molecules were recently described to be expressed on the surface of chronic lymphocytic leukemia cells and induced impaired actin polymerization at the T cell IS of both allogeneic and autologous T cells [150]. Remarkably, tumors may also express molecules on their surface that are usually expressed by immune cells to kill target cells. For instance, recent studies show that colorectal cancer cells express significant levels of the TRAIL molecule, which preferentially induces the death of CD8^+^ T cells expressing significant amounts of its receptor TRAIL-R1 [151]. Expression of this molecule would allow efficient escape of tumor cells from immune cytotoxicity by deleting activated CD8^+^ T cells.

Alternatively, the expression of inhibitory molecules on the surface of tumor cells could promote the expansion and accumulation of nonresponsive anergic T cells or CD4(+)CD25(+)Foxp3(+) T-regulatory cells (Tregs) within the tumor microenvironment (Figure 2(a)) [135, 138–140]. Regulatory T cells are mainly CD4^+^ T cells with the capacity to negatively modulate the activity of other immune cells, such as cytotoxic CD8^+^ T cells by membrane-bound and soluble molecules. For example, Tregs can negatively modulate the activity of other immune cells through the action of soluble molecules, such as IL-10, IL-35 and TGF-*β*, although the latter has been suggested to exert its activities bound at the surface of cells [152]. Noteworthy, nearly all immune cells are influenced by TGF-*β* *in vivo*, such as T cells, B cells, natural killer cells, DCs, and macrophages. TGF-*β* has been described to inhibit proliferation, differentiation and maturation of T and B cells [153–155], as well as the cytotoxicity activity of natural killer cells over tumor cells [156, 157]. Furthermore, TGF-*β* has been shown to negatively modulate the activity of antigen presenting cells, such as DCs and macrophages, decreasing their capacity to activate effector T cells [158]. Importantly, tumor cells may also secrete TGF-*β*, which contributes, among others to negatively modulate the activity of the immune cells described above, as well as promote the generation and expansion of Tregs through the conversion of immature myeloid dendritic cells into TGF-beta-secreting cells [158–162]. Another soluble molecule secreted by Tregs is IL-10, which can inhibit CD8^+^ cytotoxic T cell effector function, although indirectly through the action of APCs [163]. Contrarily, IL-35 secreted by Tregs can act directly over effector T cells [164].

Tregs may also use membrane-bound molecules to exert their negative effects over tumor immunity. For example, is has been suggested that Tregs may promote apoptosis of antitumor effector cytotoxic T cells by depleting the tumor milieu from IL-2, a T cell survival cytokine [165]. This would result from the expression of a high affinity CD25 receptor for IL-2 on the Treg surface that sequesters free IL-2 in the tumor surroundings, preventing this cytokine from reaching antitumor CTLs [165]. Membrane-bound molecules on the Treg surface could also be used to prevent efficient antigen presentation by DCs or furthermore induce inhibitory APCs termed myeloid suppressor cells (MSCs). For instance, recent data suggests that Tregs may inhibit antigen presentation on the surface of DCs by blocking MHC-II molecules thanks to the expression of LAG3, a homologous of CD4 [166, 167]. This molecule binds to MHC-II with high affinity and can induce an inhibitory signaling pathway that suppresses DCs maturation and their immunostimulatory capacity [168]. As mentioned above, CD4^+^ T cells can also exert cytotoxic functions over other cells through the secretion of granzymes and perforins. Noteworthy, Tregs can secrete granzymes and perforins to impair the function of effector T cells [169], B cells [170], and natural killer cells [169], among others. 

Thus, it is advantageous for tumors to express membrane-bound T cell inhibitory molecules, and promote the expansion of Tregs against tumor antigens to evade the immune system and persist within the host. 

## 6. Cytokines and Chemokine as Modulators of the Immunological Synapse 

Cytokines and chemokines are small proteins that play fundamental functions in immune response initiation and modulation. For T cells, cytokines and chemokines provide a wide spectrum of modulatory signals necessary for initiation, maintenance, and regulation of T cell differentiation and function (Figure 2(a)) [9, 69, 171]. Upon the onset of an immune reaction, or recall of a previous immune response, T cells can be recruited to organs with the help of chemokine gradients secreted from the affected area. Chemokines secreted from different organs can induce the expression of adhesion molecules, such as integrins and selectins, on the surface of capillary endothelium cells, which will induce T cell arrest from the blood stream to promote extravasation through vessels to the affected tissue [172]. However, chemokines also play important roles and participate in T cell-DC IS. For example, chemokines engaging the CXCR3 receptor (CXCL10, IP-10) and the CCR7 receptor (CCL19, MIP-3*β* and 6CK, SLC) have the potential to suppress T cell activation by preventing the formation of the IS [173]. Thus, gradients of these chemokines could have a negative impact over T cell activation by reducing T cell arrest over relevant surfaces [173]. Contrarily, other chemokines such as CXCL12 and CCL21 can significantly stimulate the adhesion of T cells to ICAM-1-containing planar bilayers as well as to DCs, independently of the presence of stimulating antigens [174]. Along these lines, recently Molon et al. demonstrated that during T cell stimulation, CCR5 and CXCR4 were recruited to, and accumulated at the APC-T-cell IS by a mechanism requiring chemokine secretion by APCs and chemokine receptor signaling within T cells [100]. Recruitment of chemokine receptors to the IS resulted in stronger T-cell-APC interactions, however, it reduced T cell responsiveness to other chemotactic gradients, but induced higher T cell proliferative responses and cytokine production to positive stimuli [100]. 

Because of the modulatory properties of chemokines in IS assembly and T cell migration, tumors could benefit from the expression of chemokines, in such a way to attract immune cells that negatively regulate the immune response. Consistent with this notion, CCL2 was recently shown to be expressed at the tumor site and attract myeloid suppressor cells that express the CCR2 receptor [175]. Neutralizing antibodies to CCL2 that blocked CCR2 reduced myeloid suppressor cell migration to the tumor site and reduced MSC-promoted tumor growth [175]. These data provide evidence that the CCL2/CCR2 pathway plays a pivotal role in MSC migration, which is a novel mechanism through which CCL2 promotes tumor growth [175]. Noteworthy, recently a role for chemokines in immune evasion was shown for CC-chemokine ligand 28 (CCL28) [176]. This molecule is expressed by tumors under hypoxic conditions and was shown to be able to recruit regulatory T cells to the tumor site that promotes tolerance at the tumor environment [176]. 

As mentioned above, cytokines also play key roles in T cell activation and differentiation, especially for naïve T cells that collect significant input from professional APCs through the action of soluble molecules. Studies involving cytokines and IS have led to characterizing the polarized secretion of key cytokines that modulate T cell activation, as well as clustering of receptors for these ligands at the IS. Experimental evidence suggests at least two distinct patterns of cytokine secretion by APCs and T cells: synaptic versus multidirectional [177]. For instance, the cytokines IL-2, IL-10, and IL-3 as well as IFN-*γ* were found to be secreted specifically towards the T-cell-APC IS [177]. On the other hand, cytokines such as TNF and IL-4 were found to be secreted in a multidirectional fashion [177]. This bimodal cytokine secretion enables alternative as well as enhanced cross-talk between T cells and APCs, thus establishing public and private conversations between immune cells. Concomitantly, the IFN-*γ* receptor (IFNGR) as well as IL-2R*α*, but not IL-4R, IL-6R, IL-7R, and IL-10R, were found to be localized at the IS after naïve T cell stimulation [101, 177]. Remarkably, in one study the presence of IL-4 altered the distribution of IFNGR, which in that case was no longer localized at the IS of Th cells [101]. Whether tumors alter the polarization of specific cytokines at the IS with T cells in such a way to modulate their effector functions remains to be addressed.

## 7. TCR/pMHC Binding Kinetics Governing T Cell Activation at the Immunological Synapse

**Figure 3:**
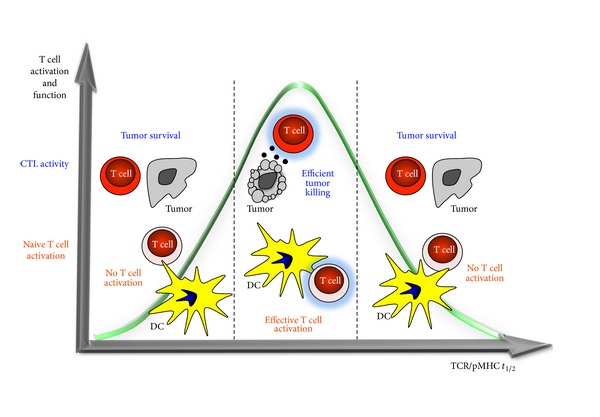
Effects of TCR/pMHC kinetics (*t*_1/2_, half-life) on T cell activation for naïve and effector T cells. TCR/pMHC interactions that are short-lived are discriminated by the TCR as nonligands in such a way to avoid the recognition of self-pMHC complexes and the activation of autoreactive T cells that may be harmful for the host (left). A model for such discrimination is termed the *kinetics proofreading model*. On the other hand, experimental data from our group supports a model for *TCR serial engagement* for T cell activation (right). That is, prolonged TCR/pMHC half-life interactions also fail to efficiently activate T cells in response to these ligands. Combining both, the *kinetics proofreading *model and the *TCR serial engagement *model, an optimal TCR/pMHC *dwell time* is required for efficient T cell activation (center). Optimal T cell activation at intermediate TCR/pMHC half-life interactions is supported by *in vitro *results using a viral peptide (naïve T cell activation) and *in vivo* data assessing either bacteria infection or tumor growth in mice (CTL activity).

T cell specificity for APCs and target cells, such as dendritic cells and tumor cells is dependent on the recognition of antigenic pMHC complexes on the surface of cells by the T cell receptor. However, pMHC complexes are not always activating and may additionally behave as either null ligands, weak agonists, or antagonists for T cells [9–11, 14, 23, 50, 178]. Remarkably, antagonists can inactivate T cells to later stimulatory ligands [10, 23]. Furthermore, certain pMHC molecules can antagonize T cell activation by interfering with the activating properties of pMHC molecules that otherwise are fully activating ligands for T cells on their own [9, 10, 15, 23]. Importantly, key parameters determining the capacity of a particular pMHC complex to promote or antagonize T cell activation are the TCR/pMHC binding kinetics and the density of pMHC complexes on the APC surface [9–11, 14, 23, 50, 178]. Contrarily to B cells, experimental evidence suggests that T cell activation is not a linear function of the duration of the TCR/pMHC interaction [10–12, 14, 50, 178]. Nonprofessional antigen presenting cells express as little as 10^5^ different pMHC complexes on their surface at any time and thus antigenic complexes will be presented at extremely low densities to T cells [179–184]. However, tumor cells as well as pathogen-infected cells display molecular evasion mechanisms to further decrease the expression of antigenic pMHC complexes on the cells surface, making the recognition of antigenic complexes by TCRs even more difficult [185–190]. Nevertheless, under these extremely scarce antigen conditions T cells ultimately manage to be activated. Experimental data suggests that T cells could be activated with as few as 1–5 antigenic pMHC complexes on the surface of an APC [191–194]. Moreover, in conditions of low ligand density a single pMHC complex on the APC surface has been suggested to be able to trigger up to 200 TCRs at the T cell surface [195]. These observations suggest that TCRs would need to be serially engaged by few antigenic pMHC complexes on the APC surface, in such a way to induce the accumulation of positive intracellular signaling within the T cell. A model that describes this notion for pMHC turnover by TCRs is known as the *TCR serial engagement model* [94, 194–197]. Importantly, this model predicts kinetics restrictions for the duration of the TCR/pMHC interaction, as prolonged TCR/pMHC interactions could hamper the serial engagement of TCRs by limiting antigenic pMHC complexes, thus jamming the accumulation of positive intracellular signaling within the T cell [10, 11]. This would be consistent with the observation that TCRs do not undergo affinity maturation and that TCR/pMHC interactions usually display 10^−6^ M affinities, unlike antibody/ligand affinities, which are within the 10^−9^–10^−12^ M range [193, 198, 199]. On the other hand, TCR/pMHC interactions that are too short-lived need to be discriminated by the TCR as nonligands, in such a way to avoid the recognition of self-pMHC complexes and the activation of autoreactive T cells that may be harmful for the host [200, 201]. A model for such discrimination has been termed the *kinetics proofreading model* [14, 20, 202–204]. Importantly, there is experimental evidence supporting both models, which furthermore have been integrated into one unifying model that suggests an optimal TCR/pMHC *dwell time* of interaction for efficient T cell activation (Figure 3) [11, 14]. In this model, both short- and long-lived TCR/pMHC interactions are inappropriate for T cell activation. pMHC ligands that induce short-lived TCR/pMHC interactions likely translate into incomplete TCR signaling, as suggested by abnormal patterns of CD3*ζ* molecule phosphorilation that would not be recognized by the T cell as fully activated (Figure 3) [205]. Similarly, this type of interactions could promote the recruitment of the SHP-1 tyrosine phosphatase, which is known to downmodulate intracellular TCR positive signaling [206]. This notion is further supported by mathematical models for IS assembly [207]. At the IS level, we have recently observed that ligands with activating TCR/pMHC half-life interactions can be competed out by ligands with short half-life interactions [23]. That is, short-lived TCR/pMHC were seen to “distract” T cells from binding TCR/pMHC ligands that bear optimal interaction kinetics [23]. Although yet not observed for tumor cells, malignant cells could avoid T cell effector functions by antagonizing T-cell activation after expressing surface ligands that produce this kind of short-lived TCR/pMHC interactions. Furthermore, we observed that short-lived TCR/pMHC interactions failed at promoting phosphorylation of signaling molecules at the T-cell-APC contact interface, which are needed for T cell activation [23]. Thus, short-lived TCR/pMHC interactions may, in some cases, not only be nonactivating, but also actively impair the activity of responding T cells [15, 23, 208–211]. Recently Yachi et al. evaluated immunological synapse formation as conjugate formation and Fluorescence Resonance Energy Transfer (FRET) analysis for CD3*ζ*-CD8*β* interactions, both for agonists and antagonists that displayed short-lived TCR/pMHC interactions [212]. Their results arose the fact that TCR-CD8 interactions are delayed for weak agonists and thus T cells may translate antigen recognition into differential recruitment of CD8 molecules to the TCR, notably at the IS [212].

To assess the effects of long-lived TCR/pMHC interactions on T cell activation, we have conducted studies with T cell lines that harbor point mutations in their TCR *β*-chains that confer variable TCR/pMHC interaction half-lives for a unique common pMHC complex [11, 19]. Interestingly, we observed that T cells that had TCRs conferring prolonged TCR/pMHC interactions failed to efficiently activate T cells in response to their ligand, thus supporting TCR serial engagement for T cell activation (Figure 3) [11]. Importantly, this observation is supported by data from other groups [14, 211, 213–216]. In our experimental settings, reduced T cell activation for long-lived TCR/pMHC interactions was abolished at higher ligand concentrations on the surface of antigen presenting cells [11]. This observation highlights the fact that optimal T cell activation is constrained to a narrower window for the duration of the TCR/pMHC interaction at low ligand density, which likely occurs for tumors (Figure 3). Furthermore, it reinforces the requirement for TCR serial engagement of pMHC molecules for T cell activation at physiological densities of cognate ligand on the APC surface. Noteworthy, our T cell activation results for long-lived TCR/pMHC interactions using high ligand densities on the APC surface or plate-bound could explain why other groups have suggested linear relationships between T cell activation and prolonged TCR/pMHC interactions [217–219].

A role for TCR/pMHC binding kinetics in T cell activation has been reported by others and us for pathogenic microbes and applies to ligand modifications that either decrease or increase the duration of the TCR/pMHC interaction [14, 178, 211, 213–215, 215, 216, 216]. Evidence for a role of TCR/pMHC binding kinetics in T cell activation and function against tumors derives from studies performed by our group with an animal model for melanoma cancer. We observed that the duration of the TCR/pMHC interaction was able to differentially regulate CTL effector functions against tumor cells *in vitro *and *in vivo* [50]. Prolonged TCR/pMHC interactions decreased the expression of cytotoxic molecules, while short TCR/pMHC interactions reduced the polarization of the T cell lytic machinery toward tumor cells (Figure 3) [50]. Furthermore, intermediate TCR/pMHC interactions induced a full array of CTL effector functions consisting of the expression of cytotoxic molecules, efficient polarization of lytic machinery towards the target cell and subsequent release of toxic granules by CTLs that killed tumor cells (Figure 3) [50]. These results are consistent with previous results from our group with an animal model for bacteria infection [178]. These data support the notion that intermediate-lived TCR/pMHC interactions are optimal for efficient CTL activity against tumors and thus the search of high affinity tumor ligands for T cells should be carefully considered [220, 221]. 

## 8. Targeting the IS for Enhancing Antitumor Immunity

**Figure 4:**
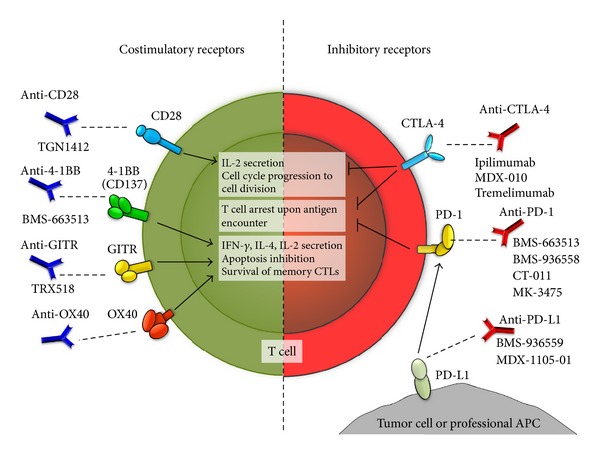
Novel therapeutic approaches targeting the immunological synapse to enhance antitumor immunity. Left. Costimulatory molecules either constitutively expressed (e.g., CD28) or inducible (e.g., 4-1BB, GITR, and OX40) that have or are being considered as targets for antitumor immunotherapy due to their positive effects on T cells after engagement (central white boxes). Right. Inhibitory molecules that have been shown to play roles in the suppression of antitumor T cells and that are expressed either at the surface of T cells (e.g., CTLA-4, PD-1) or at the surface tumor cells and professional APCs (e.g., PD-L1). Blunt arrows indicate the physiological processes (white boxes) that are affected upon the engagement of these molecules. The names of different monoclonal antibodies in present or past clinical evaluation are indicated below each antibody.

Because inhibitory molecules expressed on the surface of antigen presenting cells, such as dendritic cells and tumor cells, can deliver inhibitory stimuli at the immunological synapse that dampen the antitumor activity of T cells, these molecules are attractive targets for promoting T cell immunity against tumors. Recent novel monoclonal antibodies that either block inhibitory molecules or, alternatively, trigger costimulatory molecules on the T cell surface are being considered for therapeutic use in cancer patients (Figure 4). 

 A monoclonal antibody that blocks the T cell inhibitory molecule CTLA-4 is Ipilimumab, a recently FDA-approved antibody intended for treating advanced stages of melanoma ([222] and http://www.fda.gov/). Treatment with this antibody has been shown to significantly improve the life expectancy of patients with skin cancer and has strengthen the notion that CTLA-4 is as master regulator in controlling T cell activation (Figure 4) [223–232]. By neutralizing CTLA-4 function on early activated cytotoxic and helper T cells, Ipilimumab favors the binding of B7-1/CD80 and B7-2/CD86, that are expressed on the APC surface to the activating molcule CD28 on the T cell membrane [233, 234]. Thus, blocking CTLA-4 with Ipilimumab not only abrogates negative signaling in T cells, but also promotes their activation by allowing the activating molecule CD28 to be engaged by B7 ligands [235]. The mechanisms underlying enhanced tumor rejection after CTLA-4 blockade have been evidenced, in part from animal studies using microbe infection. One study using *Listeria monocytogenes *showed that a single dose of a mouse anti-CTLA-4 antibody was able to boost primary cytotoxic CTL responses and enhance the expansion of specific memory CD8^+^ T cells [236]. Another study using *Leishmania donovani,* also an intracellular pathogen, demonstrated that early after infection CTLA-4 blockade potentiated the expression of IFN-*γ*, IL-4, and proinflammatory chemokine, which favor microbe clearance [237]. Because CTLA-4 interferes with proximal TCR signaling by affecting the segregation of ZAP-70 into microclusters and activates phosphatases such as SYP, blocking its activity would likely favor TCR engagement and the assembly of activating ISs [238–240]. Due to the favorable results with Ipilimumab in early clinical trials with melanoma patients, this antibody is now being tested in ongoing clinical trials on patients bearing other solid tumors ([241] and http://clinicaltrials.gov/).

 Another T cell inhibitory molecule that could be targeted to treat tumors is PD-1, which binds PD-L1 and PD-L2 expressed on the surface of APCs and tumor cells [242–244]. Indeed, *in vitro* studies have shown that blocking PD-1 can significantly boost the expansion of antitumor CTLs that target the melanoma antigen MART-1 [245]. Blocking PD-1 *in vivo* in a mouse model for melanoma tumors resulted in improved CTL responses, although tumor growth was reduced when compared to the anti-CTLA-4 mono-therapy [246]. However, these results have encouraged the initiation of several clinical trials aimed at evaluating PD-1 blockade in patients with advanced stage cancers ([241] and http://clinicaltrials.gov/) (Figure 4). Because DCs have been shown to modulate peripheral tolerance to self-constituents by mechanisms that depend both on CTLA-4 and PD-1 engagement on the T cell surface, it is likely that positive synergisms could emerge between Ipilimumab and anti-PD-1 treatments to boost antitumor immunity [247]. Along these lines, additive effects have been observed for combined CTLA-4/PD-1 blockade in a B16 mouse melanoma model [248]. Here, CTLA-4/PD-1 blockade led, among others, to increased infiltration and cytotoxicity of melanoma-specific CD8^+^ T cells into the tumors [248]. Thus, blocking the activity of inhibitory molecules on the T cell surface, which dampen their antitumor activity, is an attractive strategy to enhance antitumor immunity. Ongoing clinical trials testing these approaches will hopefully provide new therapeutic alternatives to treat tumors [241]. 

Another strategy aiming to activate tumor-specific T cells is triggering constitutive and inducible costimulatory molecules expressed on the surface of T cells using agonistic monoclonal antibodies. Such antibodies would mimic the function of activating receptors on the APC, such as B7 receptors, which bind CD28, among others (Figure 4). Indeed, transfection-induced expression of B7 receptors on melanoma cells has been shown to increase CTL-mediated cytotoxicity *in vivo* [249]. Treating animals in a mouse melanoma model with CTLs stimulated *ex vivo* with an anti-CD28 antibody also provided promising results with increased CTL activity and tumor clearance [250]. These observations promoted the assessment of a CD28 agonistic antibody (TGN1412) in humans to treat tumors [251]. However, intravenous administration of the antibody unexpectedly induced a systemic inflammatory response, known as a cytokine storm, which was severely deleterious for otherwise healthy individuals [251]. This unanticipated outcome underscored the need for selecting better targets for T cell activation, such as inducible rather than constitutively expressed costimulatory molecules on the T cell surface. Current novel approaches have focused on members of the TNF superfamily as safer alternative targets due to their selective expression on activated T cells [252]. Such molecules include OX40, 4-1BB, and GITR, which have been implicated in key processes of T cell activation and differentiation, mainly acting as redundant molecules that modulate the survival and effector functions of antigen-activated T cells (Figure 4) [253]. OX-40 has been identified as critical for *in vivo* priming and imprinting of effector functions in T cells, such as IFN-*γ*, IL-2, and IL-4 secreting functions of antigen-specific T cells [254–256]. On the other hand, 4-1BB has been associated with maintenance of antigen primed T cells and memory CD8^+^ T cells *in vivo* along with increased *in vitro *proliferation and increased resistance to Treg cell suppression for both, CD4^+^ and CD8^+^ T cells [257–259]. Similarly, GITR expression on CD8^+^ T cells has been shown to be required for clonal expansion after T cell priming, and for conferring effector T cell resistance to Treg-induced suppression [260–262]. Indeed, activation of OX40, 4-1BB, and GITR with activating antibodies in animal models for tumor growth has proven to positively stimulate antitumor T cells and reduce the induction of Tregs that dampen antitumor immunity (Figure 4) [263–269]. Treating animals with an anti-GITR antibody (DTA-1) significantly impaired intratumor Treg accumulation without altering systemic Treg frequencies nor abrogating the intrinsic suppressive activity of Tregs within the tumor-draining lymph nodes [270]. This effect resulted in a greater Teff : Treg ratio in the tumor and enhanced tumor-specific CD8^+^ T cell activity [270]. However, to be effective against tumors, antibody-mediated triggering of GITR seems to require T cells to secrete IFN-*γ* [271]. Similarly, agonist antibodies to OX40 have been shown to functionally inactivate Tregs at the tumor site [267, 272]. However, agonist antibodies to OX40 were also shown to increase the proportion of CD8^+^ T cells at the tumor site in animal tumor models and increase their antitumor function [272]. Furthermore, engaging OX40 caused significant changes in the tumor stroma by decreasing the number of macrophages and myeloid-derived suppressor cells and decreasing the expression of transforming growth factor beta [267]. OX40 engagement has also been seen to induce increased numbers of infiltrating DCs migrating to draining lymph nodes, which is thought to generate a new wave of tumor-specific cytotoxic T lymphocytes [272]. Finally, animals treated with an agonist antibody to 4-1BB have been shown to induce high levels of CD8^+^ tumor-specific T cells and efficient antitumor immune response *in vivo,* which was mediated, in part, by increased survival of effector and memory CD8^+^ T cells upon activation [273]. Thus, engaging 4-1BB can significantly enhance CTL-mediated tumor clearance. Overall, these results have led to the assessment of such antibodies in clinical trials, which are currently ongoing to test their effects in humans bearing different types of cancer (Figure 4) [241].

## 9. Concluding Remarks

The IS plays a pivotal role in defining immunity to tumors. At the IS assembled between T cells and professional antigen presenting cells, such as DCs, membrane-bound and soluble molecules define the outcome of T cells, either towards activated or regulatory phenotypes. Despite the fact that T cell with effector functions against tumors are desired for tumor clearance, tumors can evolve molecular mechanisms to impair this outcome. Indeed, tumors can target T cells to promote their differentiation into regulatory T cells, as a means to downmodulate their activity and that of other antitumor immune cells. By expanding Tregs, tumors are likely to escape more efficiently from the effects of cytotoxic T cells, B cells, and natural killer cells, altogether perpetuating an inhibitory *milieu* at the tumor site that also dampens macrophage and dendritic cell function. This process is also favored by cytokines and chemokines released at the tumor site. Remarkably, tumors have been shown to adopt some immunomodulatory molecules, that were thought to be mainly restricted to professional APCs and express them at the cytotoxic IS to inhibit T cell function. By using these molecules, tumor cells have been shown to be able to directly inhibit the action of immune cells, such as CTLs. 

Noteworthy, TCR/pMHC kinetics also plays a key role in the T cell response to tumors, notably the TCR/pMHC interaction half-life. Some groups have concentrated efforts on developing tumor-derived ligands with increased affinity for TCRs to increase the reactivity of these cells to tumor antigens. Nevertheless, recent data suggests that such approach could be pointless for tumor immunity as T cell activation seems not to increase with longer TCR/pMHC interaction half-lives. In fact, this is likely detrimental for T cell activation as prolonged TCR/pMHC interactions are nonactivating [10–12, 14]. Noteworthy, such an effect would be more notorious at low antigen densities on the APCs, a common scenario for tumors. 

 Taken together, the IS is a key step for the activation and effector functions of T cells against tumors. New strategies are now being developed that either block inhibitory molecules at the IS or engage activating receptors at this structure with promising results in animal models and hopefully positive outcomes in humans. Importantly, assessment of such strategies has arisen from our increasing knowledge on the immunological synapse and how this supramolecular structure modulates and shapes T cell immunity.
